# Reconstruction of an iatrogenic anterior conchal defect with a revolving-door flap

**DOI:** 10.1186/s12893-020-01020-2

**Published:** 2021-01-06

**Authors:** Xiaofeng Liu, Tongkui Zhou, Tianlan Zhao, Zhicheng Xu

**Affiliations:** 1 Department of Plastic and Cosmetic Surgery, Xuzhou Central Hospital Affiliated to Medical Shool of Southeast University, No. 199, South Jie Fang Rd., Xuzhou, 221009 China; 2 Department of Plastic and Cosmetic Surgery, The Second Affiliated Hospital of Soochow University, No. 1055, San Xiang Rd., Suzhou, 215004 China; 3 Department of Plastic and Reconstructive Surgery, Shanghai 9th People’s Hospital, Shanghai JiaoTong University School of Medicine, No. 639, Zhi Zao Ju Rd., Shanghai, 200011 China

**Keywords:** Conchal defect, Revolving-door flap, Subcutaneous pedicle

## Abstract

**Background:**

Auricular concha has been widely used as a supporting material in rhinoplasty or repairing of auricular defects. However, complications, trauma or iatrogenic excision often result in concha defects which destroy the normal structure of the external ear and further influence daily life. Local flaps are often applied to repair the defects because of their safety and satisfactory functional and aesthetic results.

**Case presentation:**

We report a 24-year-old female who presented with a concha defect that resulted from a complication of concha cartilage graft for rhinoplasty. The anterior concha defect was covered by a revolving-door (RD) flap as a single-stage procedure. The aesthetic and functional outcomes were satisfactory at 6 months post operation.

**Conclusion:**

We recommend the RD flap as an excellent choice for conchal defect reconstruction. Satisfactory aesthetic and functional results can be achieved by this easy-to-learn technique in relatively short surgical time.

## Background

The auricular concha, located in the central part of the external ear, is of great importance in maintaining the normal structure of the external ear. This cavity is also an extension of the external auditory canal and ensures the ability to wear earphones or hearing aids. The concha contains a considerable amount of cartilage, which has been commonly used as a supporting material in rhinoplasty or the repair of local auricular defects [1–3]. Moreover, the scar at the donor site almost invisible, and there is no obvious influence on the whole structure of the auricle following concha cartilage graft harvesting. However, complications often occur, including haematoma, infection, cartilage exposure, necrosis and perforation [4]. Moreover, trauma or iatrogenic excision of a neoplastic tumour also results in concha defects [5]. Thus, different methods have been introduced to reconstruct anterior concha defects [6–9]. Local flaps are often recommended because of their safety and satisfactory functional and aesthetic results [10]. In this article, we use a revolving-door (RD) flap to cover a concha defect that resulted from a complication of concha cartilage graft for rhinoplasty.

## Case presentation

**Fig. 1:**
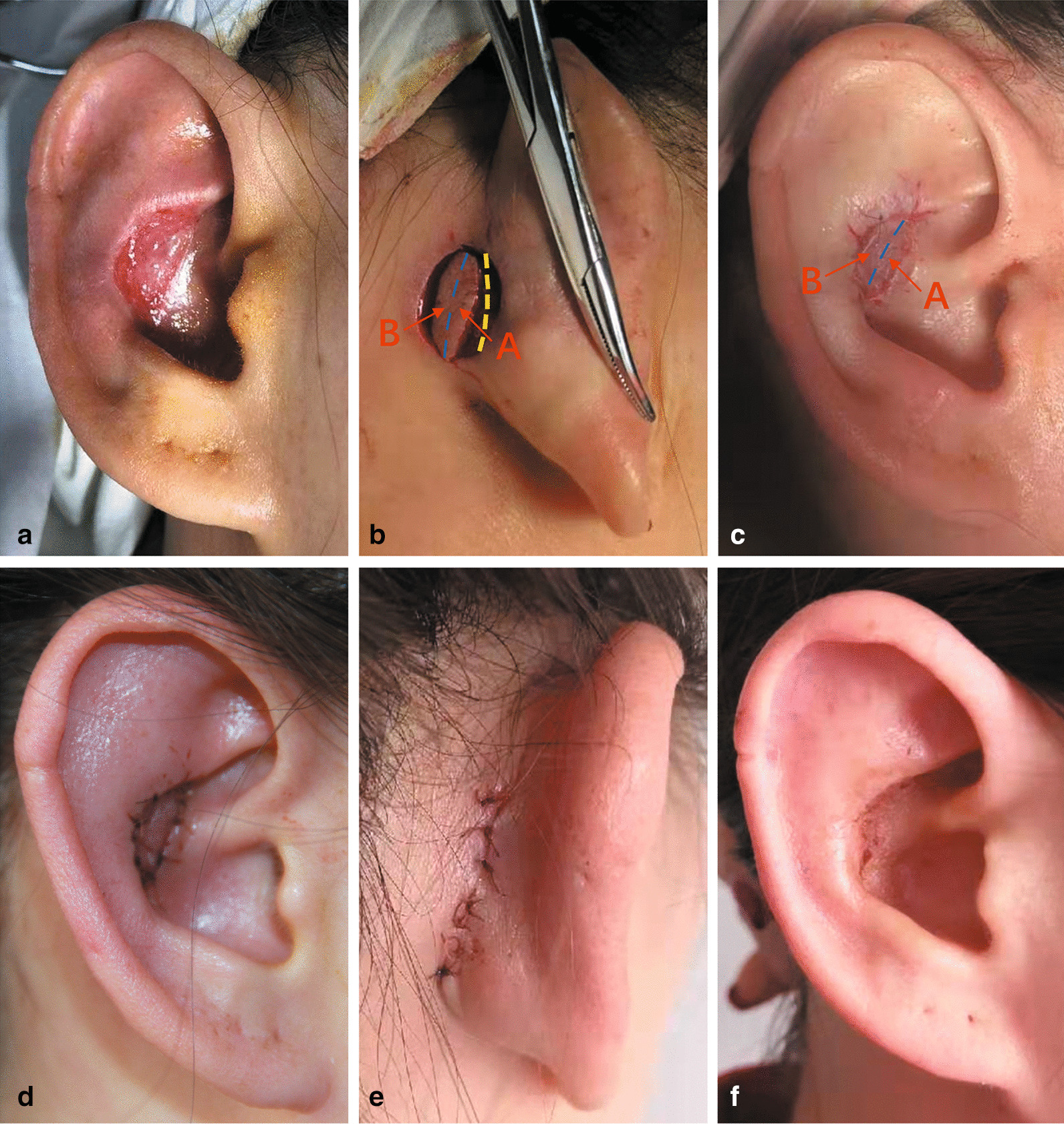
A 24-year-old female presented with an anterior conchal skin and cartilage defect after rhinoplasty with a conchal cartilage graft. A revolving-door (RD) flap was performed to cover the anterior conchal defect as a single-stage procedure. **a** Preoperative view of the anterior conchal skin and cartilage defect. **b** Intraoperative view: the flap, which was divided into retroauricular (A, red arrow) and mastoid (B, red arrow) parts by the cephalon-auricular sulcus (dashed blue lines), was dissected around the periphery and elevated along a deep plane. Its base was left unattached at the cephalon-auricular sulcus to maintain the completeness of the vascular pedicle (beneath dashed blue lines), which contained tributaries of the posterior auricular artery (PAA). A through-and-through opening space was ready to be created for transposition of the flap, as shown by the dashed yellow lines. **c** Parts A and B of the flap were rotated approximately 180° and 30° on its vertical axis, respectively, and pulled through the defect from the distal edge to cover the anterior conchal defect. Part A (red arrow) of the flap settled at the medial half of the conchal defect, and part B (red arrow) covered the lateral half of the defect. **d** Oblique view of the concha one day postsurgery. The flap survived completely, without total or partial failure. **e** Posterior view, one day postsurgery. The donor site was closed with interrupted mattress sutures. **f** Results 6 months after the surgery. There were no obvious scars or retraction at the recipient site

An anterior conchal skin and cartilage defect occurred in a 24-year-old female patient after she underwent rhinoplasty with a concha cartilage graft in another clinic. She started to feel pain in her right ear 2 days after rhinoplasty and then received antibiotic injections for 5 days. However, the pain only intensified. When the bolster was removed 7 days post operation, a 2.5 × 1.5 cm full-thickness skin and cartilage defect was observed in the anterior surface of the concha. Mismanagement of the tight dressing was considered the cause of the iatrogenic conchal defect. Before defect reconstruction, the necrotic tissue was debrided surgically, and the wound was dressed for another 1 week (Fig. 1a). Thereafter, a revolving-door (RD) flap was performed to cover the anterior concha defect as a single-stage procedure.

### Surgical technique

**Fig. 2:**
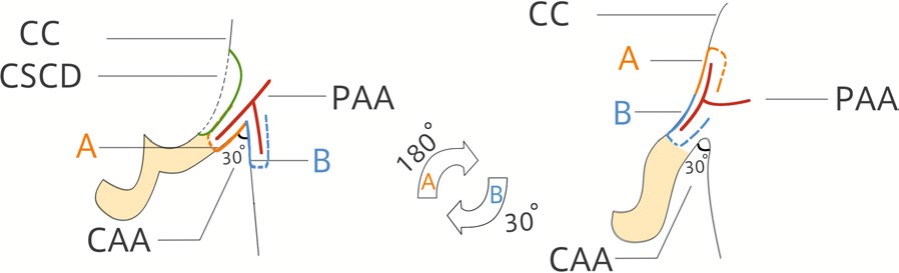
Diagram of the postauricular island pedicle flap (revolving door) and main vascular pedicle (branch from the posterior auricular artery). *PAA* posterior auricular artery, *CSCD* conchal skin and cartilage defect, *CAA* cephalo-auricular angle, *CC* conchal cavity

The patient was in the supine position, and local anaesthesia was administered via an injection of lidocaine with 1:100 000 epinephrine. The RD island flap mainly included the retroauricular and mastoid subcutaneous tissue and skin and the subcutaneous pedicle. As shown in Figs. 1b, c and 2, the size of the flap was 2.5 × 1.5 cm based on the size and shape of the anterior conchal defect, and the flap was divided into retroauricular (A) and mastoid (B) parts by the cephalon-auricular sulcus. The flap was dissected around the periphery and meticulously elevated along a deep plane. Furthermore, great care should have been taken to leave its base unattached at the cephalon-auricular sulcus to maintain the completeness of the vascular pedicle, which contained tributaries of the posterior auricular artery (PAA). After a through-and-through opening space was created, parts A and B of the flap were rotated 180° and 30°, respectively, from the distal edge to the vertical axis through the defect and were placed at the margin of the anterior conchal defect. Part A of the flap settled at the medial half of the conchal defect, and part B covered the lateral half of the defect. It is important to make an opening space with a proper size to allow the passage of the flap with no resistance. Meanwhile, the subcutaneous pedicle should also be kept adequately loosened, without physical constriction, to facilitate the blood supply to the skin flap. Thus, the transposed flap could settle appropriately, with no entrapment or deformation, as well as with a lower risk of skin flap necrosis. Both the flap and the donor site were closed with interrupted mattress sutures (nylon 5/0), which were removed 7 days following the surgery.

The operative time was 1 h and 30 min. The RD flap survived completely without total or partial failure (Fig. 1d, e). There were no obvious scars or retraction in the recipient or donor site. Moreover, the cephalon-auricular angles of the reconstructed auricle were similar to those on the normal side. The aesthetic and functional outcomes were satisfactory at 6 months post operation (Fig. 1f). The patient recovered with no deformities or complications.

## Discussion and conclusion

Autologous concha cartilage has been widely used in rhinoplasty and the reconstruction of partial auricular defects for dozens of years. However, some complications following conchal cartilage graft harvesting, such as haematoma, infection and skin necrosis due to mismanagement of tight dressings, may result in partial conchal defects. Other reasons include traumatic perforation, iatrogenic excision of a tumour or congenital malformation. Concerning the important functionality of the anatomical region, it is extremely necessary to restore the original shape and appearance.

Many reconstructive techniques have been introduced to reconstruct anterior conchal defects. Delayed wound healing is promising only in defect areas less than 1 cm in diameter with a complete perichondrium [6]. Split or full-thickness skin grafts could be applied to larger defects but may lead to a poor colour match, deformed contour or skin pigmentation at follow-up [7, 8]. Thus, local flaps are recommended because of their improved safety and better aesthetic results compared to skin grafts.

Periauricular flaps are the most commonly used method [9]. In 1972, Masson first illuminated the RD island flap for the reconstruction of concha-helix defects [10]. The flap is generally called the RD flap because it is elevated from the distal margin and rotated 180° on its vertical axis through the defect, similar to a revolving door. This flap is a local random flap, which is nurtured by a subcutaneous pedicle mainly supplied by the vascular anastomosis between the superficial temporal artery (STA) and the posterior auricular artery (PAA) [11, 12]. Park [13, 14] proved that even the antero-auricular surface is dominated by many branches from the PAA. Thus, great care should be taken not to damage the main stem of the PAA when dissecting the flap at the cephalon-auricular sulcus. Dessy compared the RD flap and full-thickness skin grafts (FTSGs) in the reconstruction of conchal defects [8]. The RD flap was introduced as the first choice because of its ease of use, shortened operative time, and favourable functional and cosmetic results at follow-up.

We think it is important to keep the subcutaneous pedicle when dissecting the flap. Moreover, the tissue surrounding the pedicle should be released as much as possible to ensure full extension and no compression of the flap. Meanwhile, the pedicle should be large enough to maintain circulation at the distal end of the flap. By doing so, the flap could accommodate the defect with no tension or circulation dysfunction.

Compared to skin grafts, we found that the RD flap leads to more aesthetic results with a better colour match, less retraction and less skin pigmentation. Furthermore, the influence of the donor site is limited, and only a one-stage operation is needed. Therefore, this approach reduces the complications, operation time and cost.

In this case, we recommend that the RD flap is an excellent choice for iatrogenic conchal defect reconstruction. Satisfactory aesthetic and functional results were achieved by this easy-to-learn technique in relatively short surgical time.

## Data Availability

The datasets used and/or analysed during the current study available from the corresponding author on reasonable request.

## Abbreviations

RD: Revolving-door
PAA: Posterior auricular artery
STA: Superficial temporal artery
FTSGs: Full-thickness skin grafts PAA
CSCD: Conchal skin and cartilage defect
CAA: Cephalo-auricular angle
CC: Conchal cavity
